# Quality of blood culture testing - a survey in intensive care units and microbiological laboratories across four European countries

**DOI:** 10.1186/cc13074

**Published:** 2013-10-21

**Authors:** Roland PH Schmitz, Peter M Keller, Michael Baier, Stefan Hagel, Mathias W Pletz, Frank M Brunkhorst

**Affiliations:** 1 Center of Clinical Studies, Jena University Hospital, Salvador-Allende-Platz 27, 07747 Jena, Germany; 2 Paul-Martini Sepsis Research Group, Jena University Hospital, Salvador-Allende-Platz 27, 07747 Jena, Germany; 3 Institute of Medical Microbiology, Jena University Hospital, Erlanger Allee 101, 07747 Jena, Germany; 4 Department of Gastroenterology and Hepatology, Jena University Hospital, Erlanger Allee 101, 07747 Jena, Germany; 5 Center of Infectious Diseases and Hospital Hygiene, Jena University Hospital, Erlanger Allee 101, 07747 Jena, Germany; 6 Center of Sepsis Control and Care (CSCC), Jena University Hospital, Salvador-Allende-Platz 27, 07747 Jena, Germany; 7 Department of Anesthesiology and Intensive Care Medicine, Jena University Hospital, Erlanger Allee 101, 07747 Jena, Germany

## Abstract

**Introduction:**

Blood culture (BC) testing before initiation of antimicrobial therapy is recommended as a standard of care in international sepsis guidelines and has been shown to reduce intensive care unit (ICU) stay, antibiotic use, and costs in hospitalized patients. Whereas microbiological laboratory practice has been highly standardized, shortfalls in the preanalytic procedures in the ICU (that is indication, time-to-incubation, blood volume and numbers of BC sets) have a significant effect on the diagnostic yield. The objective of this study was to gain insights into current practices regarding BC testing in intensive care units.

**Methods:**

Qualitative survey, data collection by 138 semi-structured telephone interviews in four European countries (Italy, UK, France and Germany) between September and November 2009 in 79 clinical microbiology laboratories (LABs) and 59 ICUs.

**Results:**

Whereas BC testing is expected to remain the gold standard for sepsis diagnostics in all countries, there are substantial differences regarding preanalytic procedures. The decision to launch BC testing is carried out by physicians vs. ICU nurses in the UK in 92 vs. 8%, in France in 75 vs. 25%, in Italy in 88 vs. 12% and in Germany in 92 vs. 8%. Physicians vs. nurses collect BCs in the UK in 77 vs. 23%, in France in 0 vs. 100%, in Italy in 6 vs. 94% and in Germany in 54 vs. 46%. The mean time from blood collection to incubation in the UK is 2 h, in France 3 h, in Italy 4 h, but 20 h in German remote LABs (2 h in in-house LABs), due to the large number of remote nonresident microbiological laboratories in Germany. There were major differences between the perception of the quality of BC testing between ICUs and LABs. Among German ICU respondents, 62% reported that they have no problems with BC testing, 15% reported time constraints, 15% cost pressure, and only 8% too long time to incubation. However, the corresponding LABs of these German ICUs reported too many false positive results due to preanalytical contaminations (49%), insufficient numbers of incoming BC sets (47%), long transportation time (41%) or cost pressure (18%).

**Conclusions:**

There are considerable differences in the quality of BC testing across European countries. In Germany, time to incubation is a considerable problem due to the increasing number of remote LABs. This is a major issue of concern to physicians aiming to implement sepsis guidelines in the ICUs.

## Introduction

Blood culture (BC) testing before initiation of antimicrobial therapy is recommended as a standard of care in international sepsis guidelines [1] and has been shown to contribute to a decrease in ICU stay [2-4]. Furthermore, BC testing is one of the cornerstones for antibiotic stewardship programs, which has been shown to reduce antibiotic overuse and costs in hospitalized patients [5,6].

Beside limitations of BC testing, for example antibiotic/antimycotic treatment prior to sampling, low proportion of causative agents in the blood samples, and frequent fastidious or noncultivable organisms [7-9], a high degree of standardization in microbiological laboratory (LAB) practice warrants for an overall positivity of approximately 30 to 40% in case of severe sepsis or septic shock [10]. In a recent large multicenter trial from Germany [11] 33% of patients with severe sepsis or septic shock had proven bacteremia. This is in contrast to a rate of only 9.6% of positive blood cultures observed in clinical practice in German ICUs aside from protocolized care [12] and underlines shortfalls in the preanalytic procedures in the ICU. Such shortfalls cover inadequate skin antisepsis and sampling techniques, as access via intravenous catheters, low blood volumes and low numbers of BC sets drawn for inoculation, prolonged time to incubation, suboptimal preincubation prior to automated cultivation at 37°C, which have a significant effect on the diagnostic yield [13-15].

The numbers of BC sets processed per hospitalized patients are off particular importance. According to the case mix of the hospital, inoculation of 100 to 200 BC sets per 1,000 patient days is recommended [16,17]. These numbers are, however, far from routine use, at least in Germany, where 55 BCs per 1,000 patient days were surveyed in 201 ICUs in 2009 in contrast to France, where 165 BCs per 1,000 patient days were quoted [18]. The 2010 annual report of the European Antimicrobial Resistance Surveillance Network (EARS-Net) specified only 12.1 BCs per 1,000 patient days in 37 hospitals in Germany, compared to 46.5 in 27 hospitals in France, 46.1 in 26 hospitals in the UK, and 70.7 BCs per 1,000 patient days in 22 hospitals in Italy [19]. In a recent study published by the National Reference Centre for Hospital Infections (NRZ), data of the German hospital nosocomial infection surveillance system (KISS) from 2006 were used to investigate the association between the frequency of blood cultures and central venous catheter-associated bloodstream infection (CVC-BSI) rates in 223 intensive care units (ICU) [20]. The median number of BC sets taken was 60 with a huge variation from 3.2 to 680 per 1,000 patient days. The authors concluded that if an external benchmarking of CVC-BSI rates is intended, an adjustment according to the BC frequency is necessary.

Reasons for the disregard of current guidelines have been identified, among others, in infrastructural aspects. The number of infections confirmed by LABs closely depends on the availability of closely located LABs [12], which sets a focus for future improvements of uniform customs and recommendations and of technical procedures on the preanalytic side of BC routine. Furthermore, there may be differences in the quality of BC testing between countries since the establishment of clinical microbiology and infectious disease departments vary substantially among European countries. Especially in Germany, patient-centered clinical microbiology is only a branch of laboratory medicine [21].

The aim of this qualitative survey was to assess the current practice in BC testing in ICUs and LABs across four European countries. Issues were technical aspects of the preanalytic course and an assessment of the current practice and their quality on the basis of individual perceptions among the staff and directors of ICUs versus LABs.

## Materials and methods

Some 138 interviews were conducted between September and November 2009 in 79 microbiological laboratories (LABs) and 59 intensive care units (ICUs) in France, Germany, Italy, and UK (Table 1). Pediatric and neonatal ICUs were excluded. Interviewees were ICU directors, ICU residents, ICU nurses, LAB directors, and LAB managers. The survey was carried out by an international agency (Advention BP, London, UK) on behalf of BD Diagnostics (Heidelberg, Germany). To uncover prevalent trends in thought and opinion, the interview panel was selected to fulfill a given quota, for example per country 10 to 20 ICUs and microbiological laboratories, respectively. Furthermore, the panel had to be balanced between BD Diagnostics (49.5%) and bioMérieux (Craponne, France) (50.5%) customers. Data were collected using semi-structured techniques for example individual in-depth personal telephone interviews. The interview guide included, among others, a list of general topics and open questions such as sepsis awareness and indication for BC testing, preanalytic procedures, sample transport and preincubation, and BC processing and communication of results (see Table S1 in Additional file 1). The response rate was 100 percent, since personal interviews have the potential to overcome the poor response rates of a questionnaire survey [22]. According to the requirements of the ethics committee of Jena University Hospital (Jena, Germany), the survey needed no ethical approval.

**Table 1:** Interviewees participating in the survey

**Interviewees (n)**	**France**	**Germany**	**Italy**	**UK**	**Total**
Total (n)	39	32	30	37	138
ICUs (n)	16	13	17	13	59
LABs (n)	23	19	13	24	79
**Type of structure (%)**					
Private	10	37	0	0	12
Public	90	63	100	100	88
ICUs					
Private	0	23	0	0	5
Public	100	77	100	100	95
LABs					
Private	17	47	0	0	16
Public	83	53	100	100	84
**Interviewee position (n)**					
ICUs					
Head of ICU	3	6	0	0	9
Physician	7	7	12	12	38
Nurse	6	0	5	1	12
LABs					
LAB director	8	17	5	5	35
LAB manager	5	0	3	15	23
Microbiologist	10	2	5	4	21

LAB, microbiological laboratory.

## Results

### Sepsis awareness

Throughout all countries surveyed, sepsis and its timely diagnosis are considered as top priorities for both ICUs and LABs. Sepsis awareness is perceived as increasingly important for 46% of interviewees in the UK, 43% in Italy, and 30% in Germany, due to its high incidence and mortality, and the importance of timely diagnosis for recovery. Medical staff in all countries noted increasing efforts how to detect and treat sepsis and how to implement educational programs for infection control in their hospitals. In the UK, critical care outreach teams have been established in certain hospitals in order to increase the medical staff’s awareness throughout the hospital [23].

### Indication for BC testing

All interviewees claimed that in their institutions BCs are collected and broad-spectrum antibiotics are administered immediately, if sepsis is suspected clinically. In general, the four systemic inflammatory response syndrome (SIRS) criteria of body temperature (fever (≥38°C) or hypothermia (≤36°C)), heart rate (tachycardia ≥90 heartbeats per minute), respiratory rate (tachypnea ≥20 breaths/minute or hyperventilation pCO_2_ <36 mmHg), and white blood cell count (leucocytosis (>12,000 cells/μl), leucocytopenia (<4,000 cells/μl), presence of immature neutrophiles) are monitored. The presence of one suspicious sign (especially the presence of fever) is usually sufficient to launch a BC. If more than one sign is present, a systematic workup is initiated. Further standard cultures (for example, urine, tracheal specimen, wound, cerebral fluids, and other swabs) are regularly performed.

### Preanalytic procedures

#### Numbers of BCs cultured

**Figure 1:**
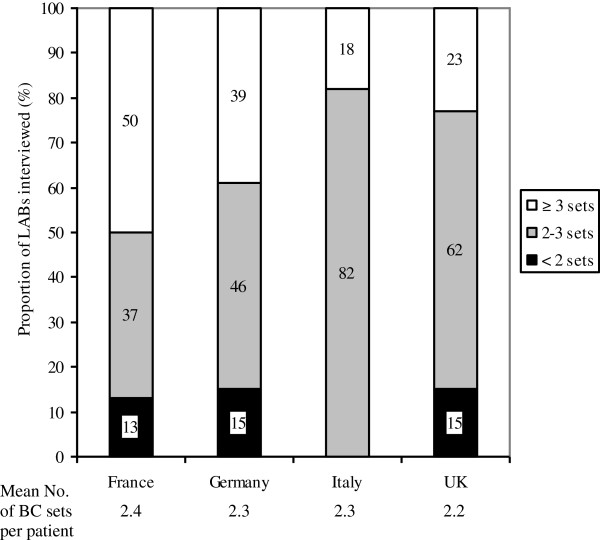
**BCs processed by 79 LABs from 59 ICUs in four European countries.** Given are mean numbers of BC sets processed in the LABs per patient within 24 hours and the average number of BC sets taken per patient in the ICUs (below). BC, blood culture; LAB, microbiological laboratory.

Most ICUs claimed to collect between two and three BC sets per patient with varying numbers by country (Figure 1). In contrast, wards collect only between 1.3 (Germany) and 1.8 (France) BC sets per patient. Less than 15% of ICUs claimed to collect less than two BC sets per patient. ICUs account for a significant proportion of BC sets processed in LABs, ranging from 15% in the UK to 33% in Germany.

#### Launch of BCs

Considerable country-specific differences were identified regarding BC collection and processing, including transportation to the LAB, timely feedback and communication procedures of results back to the ICU. While the decision to order a BC is typically taken by physicians in all countries, blood sampling is mainly carried out by physicians in the UK, by nurses in France and Italy, and by both in Germany (Table 2).

**Table 2:** Collection, transport and processing of BCs in four European countries

	**France**	**Germany**	**Italy**	**UK**
**Sample transport**				
Time to incubation (h)				
On-site LABs	3	2	4	2
Remote LABs	-	20	-	-
Cultures incubated with a delay of >8 h (%)				
On-site LABs	9	10	9	6
Remote LABs	-	>60	-	-
**Modes of transportation (%)**				
Van	36	71	23	15
Porter	32	0	77	50
Pneumatic tube	32	29	0	35
**LAB opening hours (%)**				
8 h 5 days per week	41	40	31	19
8 h 7 days per week	41	40	46	62
24 h 7 days per week	18	20	23	19
**BC management outside**				
**LAB opening hours (%)**				
Storage at room temperature	73	86	67	27
(up to 12 h delay)				
Access to BC system in the LAB	27	0	33	73
(1 h delay)				
Access to BC system in the ICU (no delay)	0	14	0	0
**Interest in relocation of BC systems into ICU (%)**				
LABs	22	47	33	21
ICUs	17	88	86	0
**Decision to launch BC (%)**				
Physician	75	92	88	92
Nurse	25	8	12	8
**Responsible for BC collection (%)**				
Physician	0	54	6	77
Nurse	100	46	94	23
**Mode of BC collection (%)**				
Intravenous catheter only	33	8	0	23
Peripheral venipuncture only	20	42	76	23
Both	47	50	24	54

BC, blood culture; LAB, microbiological laboratory.

#### Sampling technique

Techniques for blood sampling vary across countries (Table 2). A fresh peripheral venipuncture is more preferred in Germany and Italy, while blood collection via an intravenous catheter is more preferred in France and the UK. For collection, traditional systems (that is syringe and needle) or closed systems (that is winged collection sets, vacuum systems) are used in all countries. Closed systems are primarily used in France (71%), whereas Germany has the highest rate in the usage of syringes and needles (42%).

#### Blood volumes collected

**Figure 2:**
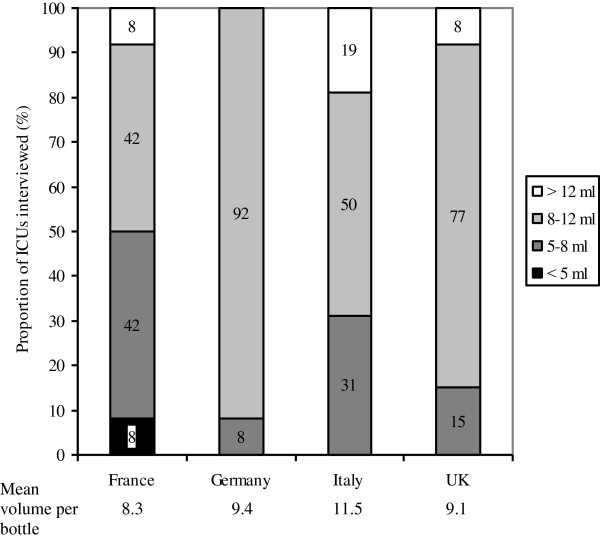
**Blood volumes collected per BC bottle in 59 ICUs in four European countries.** Given are mean blood volumes filled into BC bottles and processed in the LABs. The mean volumes per bottle are given below. BC, blood culture; LAB, microbiological laboratory.

Blood volumes collected per bottle vary between an average of 8.3 ml in France to 11.5 ml in Italy, while the majority of ICUs collect 8 to 12 ml of blood per bottle as requested by the LABs. Some 86% of the ICUs are aware that pathogen detectability is directly proportional to the amount of blood volume per bottle taken (Figure 2).

### Sample transport and preincubation

Time to incubation depends on transportation time, LAB opening hours, and BC management outside these timelines. Time-to-incubation ranges from 2 h in the UK and up to 20 h in German remote nonresident LABs (Table 2). For transportation, mainly vehicles/vans are used in Germany, where 23% of LABs are private, nonresident LABs. In Italy and in the UK transport service personnel is predominantly responsible for BC transportation within the hospital. In-house pneumatic tube systems are used in an about one-third of hospitals in France, Germany and the UK, but are not available or not used in Italy for BC transportation (Table 2).

The majority of LABs are closed overnight in all countries. Only about 40% offer services on weekends, with the exception of UK, where 62% are opened during weekends. Many LABs have on-call services for infectious emergencies. However, this service is rarely available for BC testing and management. Accordingly, the majority of BCs are stored at room temperature outside LAB opening hours, except in the UK where cultures are often preincubated in the LAB, which is served by the transport service personnel. Due to the high number of private nonresident LABs in Germany, 14% of German ICUs have established a local BC incubator device in order to shorten time to incubation. Remarkably, 88% of German and 86% of Italian ICUs are interested in the relocation of the BC incubation device at their ICUs. This is also supported by 47% of German and 33% of Italian LABs. The interest is considerably lower in the UK (ICUs: 0%, LABs: 21%) and in France (17%/22%) (Table 3).

**Table 3:** Major challenges regarding BC testing in sepsis routine identified in 79 ICUs and 59 LABs across four European countries

**Challenges (%)**	**France**	**Germany**	**Italy**	**UK**
**ICUs**				
No challenges	50	**62**	18	38
Time constraints	19	15	**41**	8
Cost pressure	0	15	**41**	15
Insufficient training of personnel	0	0	18	**31**
Excessive time to transport	0	8	**12**	0
Poor communication with LAB	**13**	0	6	0
Other	**25**	0	6	8
**LABs**				
No challenges	19	31	18	**46**
Excessive time to transport	4	**37**	23	0
Insufficient incoming sample volumes/number of BC sets	**43**	42	0	21
Cost pressure	9	16	**54**	29
Mislabeling of BC bottles	13	0	**23**	4
Many false negatives	9	**21**	8	17
Many false positives due to				
Inappropriate taking of blood samples	**61**	53	0	38
Delayed transport to the LAB	**9**	0	8	0
Low reactivity of clinicians	0	**11**	0	4

BC, blood culture; LAB, microbiological laboratory.

### BC processing, report of results and communication strategies

**Figure 3:**
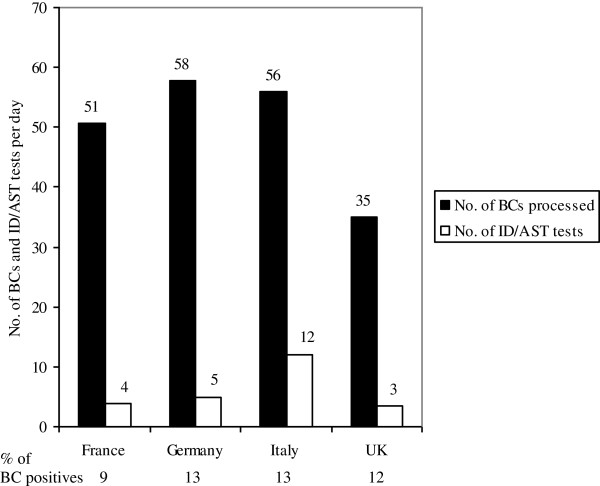
**Number of BC sets processed and ID/AST tests performed per day (mean) in microbiological LABs in four European countries.** The mean percentage of positive BC sets processed per day and LABs are given below. BC, blood culture; ID/AST, identification and antibiotic susceptibility testing; LAB, microbiological laboratory.

On average, LABs process 50 BC sets per day, ranging from 35 in the UK to 58 in Germany with a positivity rate of 12 to 13%. However, identification and antibiotic susceptibility testing (ID/AST) is not performed on all positive cultures (9% in France, 13% in Germany and Italy and 12% in the UK) (Figure 3).

Positive culture results are usually communicated over the phone across all countries, while ID/AST results are communicated to the physicians only in the UK, France, and Italy. Negative results are poorly communicated immediately, but are sent out as a written report at the end of the analysis. The quality of interaction between the LAB and the ICU is perceived as very good in all countries except in Germany, where microbiologists complain about the poor reactivity of clinicians, when positive BCs require discussion and some German ICU physicians complain about the poor quality of communication with LABs, leading to delayed or incomplete transmission of results (Table 3).

In general, perceptions vary substantially between ICU physicians and LABs (Table 3). Some 42% of ICU physicians do not see any challenges in BC testing, compared to 29% of LAB physicians, who address several severe limitations in BC testing, especially in Germany.

LABs acknowledge the insufficient incoming number of BC sets and blood volumes (27%), the high rate of false positives due to non-proper skin antiseptics and collection via intravenous catheters (38%), and the cost pressure, limiting the type and number of BC sets (27%). Cost pressure is a major challenge in Italy, where 41% of ICUs and 54% of LABs agree upon this limitation.

Excessive time to transport from the ICU to the LAB is a major challenge especially in Germany and Italy (37% and 23%). Germany and France have the highest rates in insufficient numbers of BC sets and low blood volumes taken (42% and 43%) and the highest rates of false positive BCs due to inappropriate taking of blood samples (53% and 61%).

Notably, in the UK, LABs have a strong role in the decision to initiate antibiotic treatment, while in France and Germany ICU physicians are more responsible in their choice of antibiotics.

## Discussion

Blood culture testing is definitively the gold standard and primary test to evaluate patients with sepsis [24]. However, despite European efforts to standardize BC testing similar to the US Clinical and Laboratory Standards Institute (CLSI) guidelines [17], there are different perceptions regarding the performance of BC testing between the interviewees from four European countries in our survey.

The S2k guidelines of the German Sepsis Society (GSS) [25] (see Table S2 in Additional file 2), the Italian Progetto LaSER [26], and the Britain Saving Lives (NHS) guidelines [27] recommend ≥2 BC sets in case of sepsis suspicion, which is supported by the recent international guidelines of the Surviving Sepsis Campaign (SSC) [1], whereas the French National Society of Anaesthesia and Intensive Care (SFAR) give no recommendations.

Major challenges in BC testing are low rates of true positivity due to antibiotic pretreatment prior to blood withdrawal, suboptimal sample volume, an inadequate number of BC bottles cultured and delays in time to incubation.

In a French monocentric study, Vitrat-Hincky *et al*. found that only 45% of patients had adequate numbers of BC sets and only 13% had optimal sample volumes (that is ≥10 ml per bottle) [28]. The authors of a review on true-positive rate, contamination rate, and collected blood volume of BC bottles in five Belgian hospital laboratories found that more than one-third of the BC bottles handled were incorrectly filled, irrespective of the manufacturer of the blood culture vials [29].

In our survey, blood volumes collected per BC bottle varied considerably between countries with on average less than 10 ml per bottle (8.3 ml in France, Italy with 11.5 ml as an exception), though ICU staff is aware of the fact that BC positivity is proportional to the blood volume taken.

Differences in qualities of recommended blood sampling for BCs (number of sets and volume per bottle) may be partly explained by different responsibilities among the ICU staff. BC sampling is mainly carried out by physicians in the UK, by nurses in France and Italy, and by both in Germany.

In our survey, time to incubation of BCs ranged from 2 h in the UK and up to 20 h in German remote LABs. Limitations in transport times for BCs had been reported by Kerremans *et al.* in the Netherlands [30]. The median transport time in this study was 3.5 h, with 47% of cultures exceeding the recommended 4 h. Off-site location and type of clinical specialty were the most important predictors of long transport times. Cultures collected during weekend days or on wards at the largest distances from the laboratory were also associated with long transport times.

Considerable differences between countries were observed with regard to blood transport and storage prior to automated incubation in our survey. Delays in transport times were mainly due to different transport modes (that is, via van, porter, or pneumatic tube) and infrastructure. With Germany as an obvious exception, LABs are usually closely related to hospitals resulting in a transport time ≤4 h. Together with a general trend to store blood during closing times at room temperature, which accounts for a further delay of ≤12 h, up to 20 h time to incubation occurs in Germany. In consequence, up to 14% of German ICUs already have direct access to an on-site BC incubation device. The impact of immediate incubation of BCs delivered to the laboratory outside its hours of operation on turnaround times, antibiotic prescription practices, and patient outcomes was assessed by Kerremans *et al*. in a study from the Netherlands [31]. The authors found no difference in length of stay or hospital mortality, but immediate incubation of BCs outside laboratory hours reduced turnaround times and accelerated antibiotic switching.

Positive BC results are of paramount importance for patient management. Similarly to surgery, where the close cooperation with the pathologists of hospitals guarantees the intraoperative rapid section with immediate diagnosis within a few hours, BC results have to be considered as emergencies. It is therefore mandatory to notify a clinical professional (physician, nurse practitioner) responsible for the coordination of BC testing between LABs and ICUs.

Furthermore, since many patients are seen at an emergency department at first instance and initial BCs are taken there, it is the responsibility of the LABs to determine the location of the patient once the cultures are positive. Our survey shows that most LABs transmit preliminary results (that is, on Gram-staining behavior of the microorganisms grown in culture) via telephone, allowing clinical professionals to fine-tune the initial empiric antibiotic treatment. Final results, including ID/AST information, are mostly sent via facsimile or as a written letter report. This is due to the complex nature of the information (resistance-testing results for >10 antibiotics) and to time and cost reasons. Direct oral or face-to-face communication is established in all interviewees’ countries except Germany. However, improving communication of BC results (including negative results) have been shown to reduce antibiotic usage in neonatal intensive care units [32]. Telephone transmission of critical laboratory results may be inaccurate. However, a study by Howe *et al*. showed only minor transmission errors [33].

Our study has several limitations. First, aberrations from guidelines may notably in part be due to the general phenomenon that treatment recommendations in ICUs only poorly comply with practice recommendations: ICU directors perceive adherence to be higher than it actually is [34]. We did not perform an audit on order to assess actual practice. However, the results of this survey show that even perception of current BC practices in European ICUs is suboptimal. Second, the survey was qualitative in nature, so only semi-structured techniques with open questions were applied and respondents were not randomly selected and our findings are not representative. For instance, the proportion of BC sets processed in LABs is influenced by the case mix of ICUs. In addition, we have no quantitative data on preanalytic procedures, that is, contamination data, blood volume, and routine practice subsequent to inoculation of BC bottles. Furthermore, due to the exploratory outcome of our research, a statistical analysis was not performed and our data cannot be used to make generalizations. However, by providing insights into BC testing practices in European ICUs, our study generates ideas and hypotheses for later quantitative research. Finally, we did not assess knowledge and attitudes concerning interpretation of BC results and, more importantly, therapeutic consequences. However, guideline-based collection, processing and reporting of BCs are the cornerstones for successful patient management [35].

## Conclusions

Evidence-based blood culture testing is of utmost importance for ICU patients with suspected sepsis. Knowledge of the etiologic agent (bacteria or fungi) and their susceptibility against antimicrobials enables the clinician to initiate an appropriate antimicrobial therapy and to guide diagnostic procedures. Whereas microbiological laboratory practice has been highly standardized, shortfalls in the preanalytic procedures in the ICU (indication, timing, volume, numbers, collection of blood cultures) have a significant effect on the diagnostic yield. Implementation strategies involving all ICU staff are needed to overcome the gap between recommended best practices and national guidelines. Finally, the BC frequency per 1,000 patient days should be established as a quality indicator in ICUs.

## Key messages

•There are considerable differences in the quality of BC testing across European countries and also in the perception of the quality of BC testing between ICUs and LABs.

•Positive BC results are of paramount importance for patient management. Rapid communication of BC results has to be considered as an emergency. Implementation strategies involving all ICU staff are needed to improve BC testing.

•If an external benchmarking of CVC-BSI rates is intended, an adjustment according to the BC frequency is necessary.

## Abbreviations

BC: Blood culture; CVC-BSI: Central venous catheter-associated bloodstream infection; ICU: Intensive care unit; ID/AST: Identification and antibiotic susceptibility testing; LAB: Microbiological laboratory.

## Competing interests

The authors declare that they have no competing interests. The Paul-Martini Sepsis Research Group has been supported by unrestricted grants of BD Diagnostics, Heidelberg, Germany.

## Authors’ contributions

RPHS participated in the study concept and design, contributed to the analysis and interpretation of data, drafted the manuscript and critically revised it for important intellectual content, and provided statistical expertise. PMK contributed to the analysis and interpretation of data, drafted the manuscript and critically revised it for important intellectual content. MB contributed to the analysis and interpretation of data, critically revised the manuscript for important intellectual content, and provided administrative, technical, or material support. SH contributed to the analysis and interpretation of data, critically revised the manuscript for important intellectual content, and provided administrative, technical, or material support. MWP contributed to the analysis and interpretation of data, critically revised the manuscript for important intellectual content, and provided administrative, technical, or material support. FMB participated in the study concept and design, contributed to the analysis and interpretation of data, drafted the manuscript and critically revised it for important intellectual content, and provided statistical expertise and study supervision. All authors read and approved the final manuscript.

## Supplementary Material

Additional file 1: Table S1 Issues addressed in the interview guide. Click here for file

Additional file 2: Table S2 Guideline-based blood culture testing (according to [10]). Click here for file
