# Supplementary material for: Quality of blood culture testing - a survey in intensive care units and microbiological laboratories across four European countries
Source: Crit Care. 2013 Oct 21;17(5):R248. doi: 10.1186/cc13074 (PMC4056044; doi:10.1186/cc13074)
Supplement: Additional file 1: Table S1 — Issues addressed in the interview guide. [file cc13074-S1.doc]

**Additional file 1: Table S1:** Issues addressed in the interview guide.

Sepsis awareness and indication for BC testing

- Sepsis and timely diagnosis of sepsis. Main strengths and weaknesses of BC testing.
- Routine currently applied for sepsis detection. Guidelines employed for sepsis management and BC collection.

Preanalytic procedures

- Methods used for blood collection (syringe and needle / winged collection sets / collection from a central venous catheter / other).
- BCs collected / requested / received per day, BC sets per patient / episode.
- Launch / collection of BCs (physicians vs nurses).
- Sampling processing; sites of withdrawal / equipment (catheter vs. fresh venipuncture). Sample volume collected per bottle, number of sets. Measures to avoid false positives (e.g. thorough skin disinfection, etc.) / false negatives (e.g. timing relative to antibiotic treatment).

Sample transport and preincubation

- - Average total length of BC processing cycle, from sample taking to actionable results.
  - Mode of transport (blood samples stored for batched transport or sent to the LAB as soon as they have been taken; transport by porters, express forwarders, pneumatic tube systems; regulations; time-to-incubation).
  - LAB opening hours, storage in closing hours (preincubation at 37 °C or stored at room temperatures). Relocation of BC device to ICU to avoid delays in transport.
  - Handling of BCs from ICU / other wards in the LAB (immediate processing / processing in batches). Percentage of BCs with a delay (> than 8 and 24 hours) between blood collection and final incubation.
- ID/AST tests performed on BCs per day.

BC processing and result communication

- - Communication of positive / negative results from LAB to the ICU / ward. Frequency of communication. Responsiveness of ICUs to positive results and LAB availability if results need clarification.
