# Supplementary material for: Quality of blood culture testing - a survey in intensive care units and microbiological laboratories across four European countries
Source: Crit Care. 2013 Oct 21;17(5):R248. doi: 10.1186/cc13074 (PMC4056044; doi:10.1186/cc13074)
Supplement: Additional file 2: Table S2 — Guideline-based blood culture testing (according to [10]). [file cc13074-S2.doc]

**Additional file 2: Table S2:** Guideline-based blood culture testing (acc. to [24]).

**1. Point of time of blood withdrawal:**

- Withdrawal of in minimum two BCs prior to start of antimicrobial therapy otherwise at the end of dosing interval.
- BC should be withdrawn in parallel from 2 different sites or with a gap of several minutes.
- Time point and sampling sites have to be marked onto the data sheet.
- Drawing of double blood volumes and distribution onto 2 BC sets (4 bottles) in case of venipuncture is not recommended. Fresh puncture is obligate.

**2. Aseptic puncture:**

- Hygienic hand disinfection.
- Disposable gloves (not sterile).
- Skin disinfection (e.g., with 70% alcohol for in minimum 1 minute).
- Puncture without new vein palpation.
- Puncture of peripheral veins.
- **No** withdrawal from intravenous catheters (risk of contamination).
- Exception: withdrawal of one BC set (anaerobic/aerobic, each) from a catheter suspected for infection and from a peripheral vein.

**3. Contamination-free inoculation of BC bottles:**

- Cover removal.
- Disinfection of the septum with alcoholic solution (alcohol must not enter the bottle).
- Storage of not inoculated BC bottles at room temperature. Bottles must not be inoculated if cooled.

**4. Required blood volume:**

- 8–10 ml per bottle.
- Inoculation of the anaerobic flask at first (prevents entry of air bubbles from the tip pf the syringe), aerobic flask at second.
- Special media for pedriatric applications may be inoculated with 1–3 ml per bottle.
- BC bottles must not be ventilated; bottle barcodes should not be pasted over.

**5. Number of BCs required:**

- 1 BC consists of 2 BC flasks with aerobic and anaerobic culture media, respectively. If applicable, special media may be used (e.g., for the detection of fungi) additionally.
- Withdrawal of 2 to 4 BCs are recommended. Taking of only 1 BC is not sufficient, given that a negative result permits no preclusion of the presumed infection. A single detection of facultative pathogens (e.g., coagulase-negative *Staphylococcus* spp.) affords no certain discrimination between contamination and infection.
